# Synthesis of *N*-Substituted 5-Iodouracils as Antimicrobial and Anticancer Agents 

**DOI:** 10.3390/molecules14082768

**Published:** 2009-07-27

**Authors:** Supaluk Prachayasittikul, Nirun Sornsongkhram, Ratchanok Pingaew, Apilak Worachartcheewan, Somsak Ruchirawat, Virapong Prachayasittikul

**Affiliations:** 1 Department of Chemistry, Faculty of Science, Srinakharinwirot University, Bangkok 10110, Thailand; 2 Department of Clinical Microbiology, Faculty of Medical Technology, Mahidol University, Bangkok 10700, Thailand; 3 Laboratory of Medicinal Chemistry, Chulabhorn Research Institute, Bangkok 10210, Thailand

**Keywords:** 5-iodouracil analogs, *N*-alkylation, antibacterial, antimalarial, anticancer activities

## Abstract

This study reports the synthesis of some substituted 5-iodouracils and their bioactivities. Alkylation of 5-iodouracils gave predominately N1-substituted-(R)-5-iodouracil compounds **7a**-**d** (R = *n*-C_4_H_9_, *s*-C_4_H_9_, CH_2_C_6_H_11_, CH_2_C_6_H_5_) together with N1,N3-disubstituted (R) analogs **8a**-**b** (R = *n*-C_4_H_9_, CH_2_C_6_H_11_). Their antimicrobial activity was tested against 27 strains of microorganisms using the agar dilution method. The analogs **7a**, **7c** and **7d** displayed 25-50% inhibition against *Branhamella catarrhalis*, *Neisseria mucosa* and *Streptococcus pyogenes* at 0.128 mg/mL. No antimalarial activity was detected for any of the analogs when tested against *Plasmodium falciparum* (T9.94). Their anticancer activity was also examined. Cyclohexylmethyl analogs **7c** and **8b** inhibited the growth of HepG2 cells. Significantly, N1,N3-dicyclohexylmethyl analog **8b** displayed the most potent anticancer activity, with an IC_50_ of 16.5 μg/mL. These 5-iodouracil analogs represent a new group of anticancer and antibacterial agents with potential for development for medicinal applications.

## Introduction

**Figure 1:**
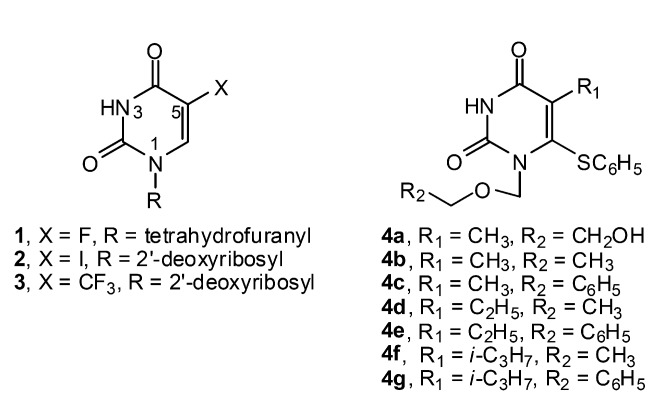
Structures of substituted uracil analogs **1**-**4** **.**

A number of pyrimidine bases have been shown to possess antiviral and anticancer activities [1], particularly uracils possessing halogens at the 5-position e.g. 5-fluorouracil; a well known anticancer drug [2], and its N1-substituted derivative **1** [3], as well as nucleoside analogs **2** and **3** of 5-iodouracil and 5-trifluoromethyluracil, which are antivirals [4]. Furthermore, acyclic-nucleoside analogs acting as anti HIV-1 agents have been reported. Examples are acyclic 5,6-disubstituted uracils **4a**-**g** (Figure 1) [4,5]. In addition, N1,N3-disubstituted uracils were reported to exhibit antibacterial and antifungal activities [6]. These N1- or N1,N3-substituted uracils were synthesized *via* alkylation of the corresponding uracils [1,6].

**Figure 2:**
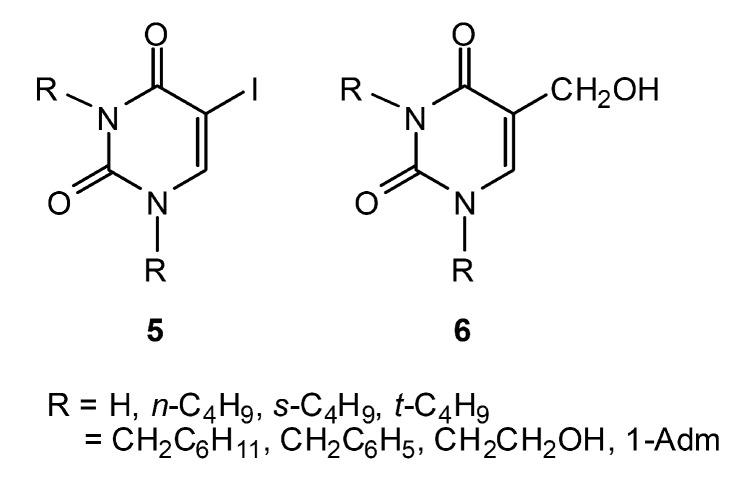
Title substituted uracils **5** and **6.**

It is known that substituted uracils (especially at the 5-position) play a vital role in many metabolic processes [7,8,9]. So far substituted 5-iodouracil and 5-hydroxymethyluracil analogs are quite rare in the literature, so there is considerable interest in searching for novel bioactive uracils with substituents at the N1 and or N1,N3 positions. The title molecules are substituted 5-iodo- and 5-hydroxymethyluracils **5** and **6** where R = alkyl and aralkyl (Figure 2). We report herein the synthesis of analogs **5** and **6** and their evaluation for antibacterial, antimalarial and anticancer actions.

## Results and Discussion

### Chemistry

The title compounds **5** were synthesized by reacting 5-iodouracil with alkyl bromides (RBr) in dimethyl sulfoxide at 80 °C for 48 h in the presence of potassium carbonate. Results are given in Table 1. It was found that alkylation of 5-iodouracil with RBr took place predominately at the N1 position when R was derived from a primary or secondary alkyl bromide to give the products 1-(1-butyl)-5-iodopyrimidine-2,4(1*H*, 3*H*)-dione (**7a**, R = *n*-C_4_H_9_, 28%), 1-(2-butyl)-5-iodopyrimidine-2,4(1*H*, 3*H*)-dione (**7b**, R = *s*-C_4_H_9_, 6.1%), 1-(cyclohexylmethyl)-5-iodopyrimidine-2,4(1*H*, 3*H*)-dione (**7c**, R = CH_2_C_6_H_11_, 28.1%) and 1-benzyl-5-iodopyrimidine-2,4(1*H*, 3*H*)-dione (**7d**, R = CH_2_C_6_H_5_, 11.4%), respectively. It was noted that primary alkyls (*n*-butyl and cyclohexylmethyl) give comparable or higher yields than aralkyl (benzyl) and higher than secondary alkyl (*s*-butyl) as follows: **7a** ≈ **7c** > **7d** > **7b.** In addition, minor N1,N3-dialkylation products: 1,3-di(1-butyl)-5-iodopyrimidine-2,4(1*H*, 3*H*)-dione (**8a**) and 1,3-bis(cyclohexylmethyl)-5-iodopyrimidine-2,4(1*H*, 3*H*)-dione (**8b**) were observed in comparable yields when R = *n*-butyl and cyclohexylmethyl, respectively. Such dialkylation of 5-iodouracil was not observed in the reaction with benzyl bromide. The reactions of 5-iodouracil with sterically hindered RBr such as R = *t*-C_4_H_9_ and 1-adamantyl (1-Adm) failed to give the products under the same conditions or when the reaction was carried out in *N,N*-dimethylformamide containing triethylamine at 140 °C for 10 h, as noted by TLC. This suggets that the *N*-alkylation proceeds *via* a S_N_2 reaction. Unfortunately, 2-bromoethanol did not react with 5-iodouracil in the presence of K_2_CO_3_ or Et_3_N as observed by TLC. *N*-Functionalizations of uracils at the N1- and N1,N3-positions were previously reported [10,11,12,13]. *O*-Alkylation of hydroxypyrimidines were also reported, e.g. 4,6-dihydroxypyrimidines gave a mixture of O4,O6- and N1,O4-disubstituted products [14]. Attempts were made to synthesize the title compound **6** under similar conditions as used for compound **5**, but this was unsuccessful.

Structures of the obtained 5-iodouracils **7** and **8** were established using ^1^H- and ^13^C-NMR, IR and mass spectra. The IR spectra showed strong CO stretching bands in the 1,651-1,716 cm^-1^ range, while the characteristic NH peak of N1 substituted uracils **7a-d** appeared in the 3,022-3,159 cm^-1^ range as sharp peaks. The ^1^H-NMR spectra showed singlets of H-6 at δ 7.53-8.17 ppm, while the C-6 peak appeared at δ 145.21-149.48 ppm in the ^13^C-NMR spectra. The HMBC spectra exhibited relationships between the H-6 proton and the carbons C-1^′^, C-2, C-5 and C-4 and conversely, of H-1^′^ with C-2, C-6 and C-2^′^. Such C-H connectivity indicated that in the uracil analogs **7a**-**d** the N1 position contained alkyl or aralkyl group substituents. Similar correlations were also observed for H-1^′′^ with C-2, C-4 and C-2^′′^, suggesting that in the case of analogs **8a** and **8b** additional substitution took place at N3. Both N1- and N1, N3-substitution patterns were in evidence when R = *n*-C_4_H_9_ and CH_2_C_6_H_11_, as found in uracils **7a**, **7c** and **8a**, **8b**, respectively. The mass spectra of analogs **7a**-**d** and **8a**-**b** all exhibited their molecular ions and base peaks resulting from fragmentations of alkyl or aralkyl at the N1- and/or N1,N3-positions, except for the analog **7a**, which showed the molecular ion as the base peak (Table 2). Based on 2D-NMR spectra (COSY, DEPT90, DEPT135, HMQC and HMBC), IR and mass spectra, the substitution patterns of the N1- and N1,N3-alkylation products were clearly identified.

**Table 1:** Alkylation products from 5-iodouracil with alkyl and aralkyl bromides.

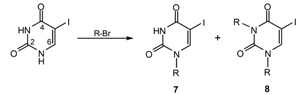			
**Entry**	**R**	**Substitution Products** (%)	
		N1-	N1, N3-
1	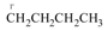	**7a** (28.0)	**8a** (6.7)
2	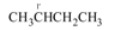	**7b** (6.1)	―
3	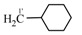	**7c** (28.1)	**8b** (7.5)
4	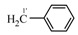	**7d** (11.4)	―
5	* t*-C_4_H_9_	―	―
6	1-Adm	―	―
7	CH_2_CH_2_−OH	―	―

**Table 2:** Selected spectral data of N1- and N1,N3-substituted uracils **7** and **8**.

**Compound**	δ (ppm)			υ_max_ (cm^-1^)			Mass spectra (*m*/*z*)	
	H-6	C-6		C=O	NH		Molecular ion	Base peak
**7a**	7.59	148.87		1715,1667	3022		294	294
**7b**	7.53	145.21		1716, 1700	3159		294	237
**7c**	7.54	149.36		1701, 1660	3159		334	238
**7d**	8.17	149.48		1714, 1669	3112		328	91
**8a**	7.60	146.74		1698, 1651	°		350	333
**8b**	7.53	147.29		1701, 1653	°		430	238

### Antibacterial activity

Antibacterial activity of the analogs 1-(1-butyl)-5-iodopyrimidine-2,4(1*H*, 3*H*)-dione (**7a**), 1-(cyclo-hexylmethyl)-5-iodopyrimidine-2,4(1*H*,3*H*)-dione (**7c**) and 1-benzyl-5-iodopyrimidine-2,4(1*H*,3*H*)-dione (**7d**) and 1,3-bis(cyclohexylmethyl)-5-iodopyrimidine-2,4(1*H*, 3*H*)-dione (**8b**) compounds and was evaluated against 27 strains of microorganisms using the agar dilution method [15]. The results (Table 3) showed that the analogs **7a**, **7c** and **7d** inhibited 50% growth of *B. catarrhalis* and 25% growth of *N. mucosa* and *S. pyogenes* at 0.128 mg/mL. However, **8b** was found to be inactive against all the tested microorganisms. The activity of such compounds has not been reported in the literature, therefore, these analogs **7a**, **7c** and **7d** are new antibacterial leads.

**Table 3:** Antibacterial activity* of substituted 5-iodouracils **7** and **8**.

Compound**	Activity	Inhibition (%)	
**7a**	Active	50^a^	25^b,c^
**7c**	Active	50^a^	25^b,c^
**7d**	Active	50^a^	25^b,c^
**8b**	Inactive	0	0

Inhibition against ^a^*B. catarrhalis*, *^b^N. mucosa*, ^c^*S. pyogenes*, *Ampicillin at 0.01 mg/mL was used as a control of the antibacterial testing system; it showed 100% inhibition on selected microorganisms (*S. aureus* ATCC 25923 and *B. subtilis* ATCC 6633). **Concentration of 0.128 mg/mL was used.

### Antimalarial activity

The activity of analogs **7a**-**d** and **8a**-**b** was tested as described [16] against *Plasmodium falciparum* chloroquine resistant (T 9.94) using chloroquine hydrochloride as a reference drug. It was found that all the tested compounds were inactive as antimalarials with IC_50_ >10^-5^ M.

### Anticancer activity

Anticancer activity assays [17] against 12 cell lines using etoposide and/or doxorubicin as positive controls were carried out. The results (Table 4) revealed that 1,3-bis(cyclohexylmethyl)-5-iodopyrimidine-2,4(1*H*, 3*H*)-dione (**8b**) was active against HepG2, A549 and HuCCA-1 with IC_50_ values of 16.5, 33.0 and 49.0 μg/mL, respectively. 1-(Cyclohexylmethyl)pyrimidine analog **7c** exhibited activity against T47D, KB, HepG2, P388 and HeLa cells with IC_50_ of 20.0, 35.0, 36.0, 41.47 and 46.0 μg/mL, respectively. In addition, **8a** inhibited the growth of MOLT-3 with IC_50_ of 37.53 μg/mL. The activity of T47D was also inhibited by **7d**, showing IC_50_ of 43.0 μg/mL. It is notable that the growth of HepG2 is selectively inhibited by 1- and 1,3-substituted cyclohexylmethyl analogs **7c** and **8b**, respectively. However, the 1,3-bis(cyclohexylmethyl) analog **8b** exhibited higher activity than 1-cyclohexylmethyl analog **7c**. This perhaps due to higher lipophilicity of 1,3-disubstituted analog **8b** which enhances its absorption by the cancer cells. Significantly, the analog **8b** was the most active against HepG2 with IC_50_ of 16.5 μg/mL. These compounds **7c**-**d** and **8a**-**b** are new potential anticancer agents. Compounds **7a** and **7b** were inactive against all the tested cell lines.

**Table 4:** Anticancer activity of substituted 5-iodouracils **7** and **8**.

Cell line	IC_50_ (μg/mL)^a,b^						
	7a	7b	7c	7d	8a	8b	Etoposide(Doxorubicin)
HepG2	>50	>50	36.00	>50	>50	16.50	12.00
HuCCA-1	>50	>50	>50	>50	>50	49.00	(0.50)
A549	>50	>50	>50	>50	>50	33.00	0.60 (0.45)
MOLT-3	NA	>50	NA	NA	37.53	>50	0.019
KB	>50	NA	35.00	>50	NA	NA	0.25
HCC-S102	>50	NA	>50	>50	NA	NA	6.00
HL60	>50	NA	>50	>50	NA	NA	0.85
P388	>50	NA	41.47	>50	NA	NA	0.12
HeLa	>50	NA	46.00	>50	NA	NA	0.38
MDA-MB231	>50	NA	>50	>50	NA	NA	0.24
T47D	>50	NA	20.00	43.00	NA	NA	0.05
H69AR	>50	NA	50.00	>50	NA	NA	30.00

NA = not tested. a: When IC_50_ >50 μg/mL denotes inactive for anticancer activity. b: The assays were performed in triplicate.

## Conclusions

Alkylation of 5-iodouracil furnished mainly N1-substituted uracils **7a**-**d**, together with minor amounts of the N1,N3-disubstituted analogs **8a**-**b**, when the substituent groups (R) were primary and secondary. Among these, **7b**-**c** and **8a**-**b** are new analogs. The analogs **7a** (R = *n*-C_4_H_9_), **7c** (R = CH_2_C_6_H_11_) and **7d** (R = CH_2_C_6_H_5_) showed 25-50% growth inhibition against *B. catarrhalis*, *N. mucosa* and *S. pyogenes* at 0.128 mg/mL. No antifungal and antimalarial activities were observed for any of the tested compounds. It is notable that anticancer activity was seen for the analogs **7c** and **8b** bearing a cyclohexylmethyl group (R = CH_2_C_6_H_11_). Significantly, the N1,N3-dicyclohexylmethyl uracil analog **8b** exhibited the most potent anticancer activity, but was inactive as an antibacterial. It can be concluded that these 5-iodouracil analogs represent a new group of anticancer and antibacterial agents with potential to be further developed for medicinal applications.

## Experimental

### General

Melting points were determined on an Electrothermal melting point apparatus (Electrothermal 9100) and are uncorrected. ^1^H- and ^13^C-NMR spectra were recorded on a Bruker AVANCE 300 NMR spectrometer (operating at 300 MHz for ^1^H and 75 MHz for ^13^C). Infrared spectra (IR) were obtained on Perkin Elmer System 2000 FTIR. Mass spectra were recorded on a Finnigan INCOS 50 and Bruker Daltonics (micro TOF) instruments. Column chromatography was carried out using silica gel 60 (0.063–0.200 mm). Analytical thin layer chromatography (TLC) was performed on silica gel 60 PF_254_ aluminium sheets (cat. No. 7747 E., Merck). Solvents were distilled prior to use. Chemicals used for the syntheses were of analytical grade. Reagents for cell culture and assays were the following: RPMI-1640 (Gibco and Hyclone Laboratories, USA), HEPES, L-glutamine, penicillin-streptomycin, sodium pyruvate and glucose (Sigma, USA), Ham’s/F12, DMEM and fetal bovine serum (Hyclone Laboratories, USA), Gentamicin sulfate (Government Pharmaceutical Organization, Thailand), 3(4,5-dimethylthiazol-2-yl)-2,5-diphenyltetrazolium bromide (Sigma-Aldrich, USA).

### Synthesis of N-substituted 5-iodouracil analogs ***7a-d*** and ***8a-b***

5-Iodouracil was dissolved in DMSO (5 mL), then K_2_CO_3_ was added and the mixture stirred at 80 °C for 15 min. Alkylating agent was added dropwise (5 min) to the solution then stirred for 48 h at 80 °C. Products were collected by filtration or by solvent extractions. Purification by silica gel column using hexane-ethyl acetate (8:2) as eluting solvent gave the required compounds. The products were recrystallized from methanol or dichloromethane-methanol (1:1).

*1-(1-Butyl)-5-iodopyrimidine-2,4(1H, 3H)-dione* (**7a**) *and 1,3-di(1-butyl)-5-iodopyrimidine-2,4(1H, 3H)-dione* (**8a**)*:* 5-Iodouracil (0.476 g, 2.0 mmol), K_2_CO_3_ (0.138 g, 1.0 mmol) and 1-butyl bromide (0.274 g, 2.0 mmol) gave **7a** (0.165 g, 25.01%) and **8a** (0.047 g, 6.67%). Compound **7a**; mp 175-176 °C; IR (KBr): υ_max_ 3,022, 2,949, 1,715, 1,667, 1,606 cm^-1^; ^1^H-NMR (CDCl_3_): δ 0.95 (t, 3H, *J* = 7.2 Hz, H-4^′^), 1.35 (sextet, 2H, *J* = 7.2 Hz, H-3^′^), 1.66 (quintet, 2H, *J* = 7.5 Hz, H-2^′^), 3.73 (t, 2H, *J* = 7.2 Hz, H-1^′^), 7.59 (s, 1H, H-6), 8.86 (br, 1H, NH-3); ^13^C-NMR (CDCl_3_): δ 13.58 (C-4^′^), 19.62 (C-3^′^), 31.19 (C-2^′^), 48.98 (C-1^′^), 67.46 (C-5), 148.87 (C-6), 150.34 (C-2), 160.31 (C-4); LRMS (EI): m/z (%) = 295 (10.97) [M + H]^+^, 294 (100.00) [M]^+^, 238 (97.07), 167 (46.60); HRMS (TOF) *m/z* [M + H]^+^ calcd for C_8_H_12_IN_2_O_2_: 294.9938 found: 294.9940. Compound **8a**; mp 70-71 °C; IR (KBr): υ_max_ 3,051, 1,698, 1,651, 594 cm^-1^; ^1^H-NMR (CDCl_3_): δ 0.93-1.01 (m, 6H, H-4^′^, H-4^′′^), 1.32-1.44 (m, 4H, H-3^′^, H-3^′′^), 1.57-1.74 (m, 4H, H-2^′^, H-2^′′^), 3.76 (t, 2H, *J* = 7.4 Hz, H-1^′^), 4.00 (t, 2H, *J* = 7.5 Hz, H-1^′′^), 7.60 (s, 1H, H-6); ^13^C-NMR (CDCl_3_): δ 13.57 (C-4^′′^), 13.69 (C-4^′^), 19.68 (C-3^′′^), 20.13 (C-3^′^), 29.50 (C-2^′′^). 31.20 (C-2^′^), 42.88 (C-1^′′^), 49.89 (C-1^′^), 67.57 (C-5), 146.74 (C-6), 150.97 (C-2), 160.05 (C-4); LRMS (EI): m/z (%) = 351 (20.33) [M + H]^+^, 350 (69.42) [M]^+^, 333 (100.00), 308 (32.97), 293 (58.48), 252 (72.27), 238 (30.76); HRMS (TOF): *m/z* [M + H]^+^ calcd for C_12_H_20_IN_2_O_2_: 351.2018 found: 351.0567.

*1-(2-Butyl)-5-iodopyrimidine-2,4(1H, 3H)-dione* (**7b**): 5-Iodouracil (2.38 g (10.0 mmol), K_2_CO_3_ 1.382 g (10.0 mmol) and 2-butyl bromide 1.372 g (10.0 mmol) furnished compound **7b** 0.18 g (6.12%). Compound **7b**; mp 194-195 °C; IR (KBr): υ_max_3159, 3034, 2962, 1716, 1700, 1654, 1599, 612 cm^-1^; ^1^H-NMR (CDCl_3_): δ 0.9 (t, 3H, J = 7.2 Hz, H-4^′^), 1.31 (d, 3H, J = 6.9 Hz, H-1^′^), 1.57-1.68 (m, 2H, H-3^′^), 4.59 (sextet, 1H, J = 6.9 Hz H-2^′^), 7.53 (s, 1H, H-6), 8.73 (br, 1H, NH-3); ^13^C-NMR (CDCl_3_): δ 10.52 (C-4^′^), 19.64 (C-1^′^), 28.69 (C-3^′^), 53.78 (C-2^′^), 67.87 (C-5), 145.21 (C-6), 150.61(C-2), 159.67(C-4); LRMS (EI): *m/z* (%) = 295 (11.18) [M + H]^+^, 294(47.12) [M]^+^, 237 (100), 167 (37.4); HRMS (TOF) *m/z* [M + H]^+^ calcd for C_8_H_12_IN_2_O_2_: 294.9938 found: 294.9941.

*1-(Cyclohexylmethyl)-5-iodopyrimidine-2,4(1H, 3H)-dione* (**7c**) *and 1,3-bis(cyclohexylmethyl)-5-iodo-pyrimidine-2,4(1H, 3H)-dione* (**8b**)*:* 5-Iodouracil 0.476 g (2.0 mmol), K_2_CO_3_ 0.138 g (1.0 mmol) and (bromomethyl)cyclohexane 0.354 g (2.0 mmol) gave **7c** 0.182 g (28.09%) and **8b** 0.065 g (7.5%). Compound **7c**; mp 240-241°C; IR (KBr): υ_max_3159, 3021, 2920, 1701, 1660, 1606, 622 cm^-1^; ^1^H-NMR (CDCl_3_): δ 0.92-1.75 (m, 11H, H-2^′^, H-3^′^, H-4^′^, H-5^′^), 3.54 (d, 2H, J = 7.2 Hz, H-1^′^), 7.54 (s, 1H, H-6), 9.04 (br, 1H, NH-3); ^13^C-NMR (CDCl_3_): δ 25.47 (C-4^′^), 26.07 (C-5^′^), 30.30 (C-3^′^), 37.35 (C-2^′^), 55.13 (C-1^′^), 67.14 (C-5), 149.36 (C-6), 150.58 (C-2), 160.38 (C-4); LRMS (EI) : m/z (%) = 335 (12.84) [M + H]^+^, 334 (76.68) [M]^+^, 252 (11.45), 238 (100.00), 208 (12.14); HRMS (TOF) *m/z* [M + H]^+^ calcd for C_11_H_16_IN_2_O_2_: 335.0251 found: 335.0252. Compound **8b;** m.p. 138-139°C; IR (KBr): υ_max_ 3075, 2924, 1701, 1653, 1617 cm^-1^; ^1^H-NMR (CDCl_3_): δ0.89-1.81 (m, 22H, H-2^′^, H-2^′′^, H-3^′^, H-3^′′^,H-4^′^, H-4^′′^, H-5^′^, H-5^′′^), 3.56 (d, 2H, J = 7.01 Hz, H-1^′^), 3.84 (d, 2H, J = 7.2 Hz, H-1^′′^), 7.53 (s, 1H, H-6); ^13^C-NMR (CDCl_3_): δ 25.51 (C-4^′′^), 25.77 (C-4^′^), 26.11 (C-5^′′^), 26.29 (C-5^′^), 30.39 (C-3^′′^), 30.76 (C-3^′^), 36.19 (C-2^′′^), 37.37 (C-2^′^), 48.80 (C-1^′′^), 56.17 (C-1^′^), 67.28 (C-5), 147.29 (C-6), 151.33 (C-2), 160.33 (C-4); LRMS (EI): *m/z* (%) = 431 (9.87) [M + H]^+^, 430 (58.49) [M]^+^, 333 (64.21), 238 (100), 97 (79.25); HRMS (TOF) *m/z* [M + H]^+^ calcd for C_18_H_28_IN_2_O_2_: 431.3237 found: 431.3242.

*1- Benzyl-5- iodopyrimidine-2,4(1H, 3H)-dione* (**7d**)*:* 5-Iodouracil 0.476 g (2.0 mmol), K_2_CO_3_ (0.138 g, 1.0 mmol) and benzyl bromide (0.342 g, 2.0 mmol) gave compound **7d** (0.075 g, 11.44%); mp 209-210°C (lit. mp 210-213°C [18]); IR (KBr): υ_max_ 3112, 3011, 1714, 1669, 1452, 1426, 733 cm^-1^; ^1^H- NMR (CDCl_3_): δ 5.01 (s, 2H, H-1^′^), 7.30-7.42 (m, 5H, ArH), 8.17 (s, 1H, H-6), 10.46 (br, 1H, NH-3); ^13^C-NMR (CDCl_3_): δ 50.86 (C-1^′^), 67.17 (C-5), 136.63 (C-2^′^),149.48 (C-6),150.79 (C-2), 160.28 (C-4), 127.88,127.97,128.73 (Ar-C); LRMS (EI): m/z (%) = 329 (5.82) [M + H]^+^, 328 (48.13) [M]^+^, 91 (100.00); HRMS (TOF): *m/z* [M + H]^+^ calcd for C_11_H_10_IN_2_O_2_: 328.9781 found: 328.9791.

### Chloroquine resistant Plasmodium falciparum (T9.94)

Human erythrocytes (type O) infected with chloroquine resistant *P. falciparum* (T9.94) were maintained in continuous culture, according to the method described previously [16]. RPMI-1640 culture medium supplemented with 25mM HEPES, 40 mg/L gentamicin sulfate and 10 mL of human serum was used in continuous culture.

### Cancer cells

Cells were grown in Ham’s/F12 medium containing 2 mM L-glutamine supplemented with 100 U/mL penicillin-streptomycin and 10% fetal bovine serum. Except HepG2 and MOLT-3 cells were grown in DMEM and RPMI-1640 medium, respectively.

### Antimicrobial assay

Antimicrobial activity of the tested compounds was performed using agar dilution method as previously described [15]. Briefly, the tested compounds dissolved in DMSO were individually mixed with 1 mL Müller Hinton (MH) broth while the negative control was the MH broth with omission of the tested compounds. The solution was then transferred to the MH agar solution to yield the final concentrations of 0.032-0.256 mg/mL. Twenty seven strains of microorganisms, cultured in MH broth at 37 °C for 24 h, were diluted with 0.9% normal saline solution to adjust the cell density of 3×10^9^ cell/mL. The organisms were inoculated onto each plate and further incubated at 37 ^o^C for 18-48 h. Compounds which possessed high efficacy to inhibit bacterial cell growth were analyzed. The microorganisms used for the activity testing are listed in Table 5.

**Table 5:** The twenty-seven strains of microorganisms used for antimicrobial activity testing.

Microorganisms	
**Gram-negative bacteria**	
***Escherichia coli ATCC 25922***	***Morganella morganii***
***Klebsiella pneumoniae ATCC 700603***	***Vibrio cholera***
***Salmonella typhimurium ATCC 13311***	***Vibrio mimicus***
***Salmonella choleraesuis ATCC 10708***	***Aeromonas hydrophila***
***Pseudomonas aeruginosa ATCC 15442***	***Plesiomonas shigelloides***
***Edwardsiella tarda***	***Xanthomonas maltophilia***
***Shigella dysenteriae***	***Neisseria mucosa***
***Citrobacter freundii***	***Branhamella catarrhalis***
**Gram-positive bacteria**	
***Stapphylococcus aureus* ATCC 25923**	***Bacillus subtilis* ATCC 6633**
***Stapphylococcus epidermidis* ATCC 12228**	***Streptococcus pyogenes***
***Enterococcus faecalis* ATCC 29212**	***Listeria monocytogenes***
***Micrococcus lutens* ATCC 10240**	***Bacillus cereus***
***Corynebacterium diphtheriae* NCTC10356**	***Micrococcusflavas***
**Diploid fungus (Yeast)**	
***Candida albicans***	

### Antimalarial assay

Antimalarial activity of the tested compounds was evaluated against *Plasmodium falciparum* chloroquine resistant (T9.94) using the literature method [16,19]. The experiments were started with synchronized suspension of 0.5% to 1% infected red blood cell during ring stage. Parasites were suspended with culture medium supplemented with 15% human serum to obtain 10% cell suspension. The parasite suspension was put into 96-well microculture plate; 50 μL in each well and then add 50 μL of various tested drug concentrations. These parasite suspensions were incubated for 48 h in the atmosphere of 5% CO_2_ at 37 °C. The percents parasitemia of control and drug-treated groups were examined by microscopic technique using methanol-fixed Giemsa stained of thin smear blood preparation.

### Cytotoxicity assays

Cytotoxicity assays were performed using the modified method described previously [17]. Briefly, cell lines suspended in RPMI-1640 containing 10% FBS were seeded at 1°10^4^ cells (100 μL) per well in 96-well plate, and incubated in humidified atmosphere, 95% air, 5% CO_2_ at 37 °C. After 24 h, additional medium (100 μL) containing the test compound and vehicle was added to a final concentration of 50 μg/mL, 0.2% DMSO, and further incubated for 3 days. Cells were subsequently fixed with 95% EtOH, stained with crystal violet solution, and lysed with a solution of 0.1 N HCl in MeOH, after which absorbance was measured at 550 nm. Whereas HuCCA-1, A549 and HepG2 cells were stained by MTT (3-(4,5-dimethylthiazol-2-yl)-2,5-diphenyltetrazolium bromide) and for MOLT-3 cell was stained by XTT. The cell lines used for the assay are listed in Table 6. IC_50_ values were determined as the drug and sample concentration at 50% inhibition of the cell growth.

**Table 6:** Twelve cell lines used for the cytotoxicity assays.

Cell lines	
Human hepatocellular liver carcinoma cell line (HepG2)	Human promyelocytic leukemia cell line (HL-60)
Human cholangiocarcinoma cancer cells (HuCCA-1)	Murine leukemia cell line (P388)
Human lung carcinoma cell line (A549)	Cervical adenocarcinoma cell line (HeLa)
T-lymphoblast (MOLT-3, acute lymphoblastic leukemia)	Hormone-independent breast cancer cell line (MDA-MB231)
Human epidermoid carcinoma of the mouth (KB)	Hormone-dependent breast cancer cell line (T47D)
Hepatocellular carcinoma cell line (HCC-S102)	Multidrug-resistance small cell lung cancer cell line (H69AR)
